# Public Submissions on the Uganda National Biotechnology and Biosafety Bill, 2012 Reveal Potential Way Forward for Uganda Legislators to Pass the Bill

**DOI:** 10.3389/fbioe.2015.00152

**Published:** 2015-10-06

**Authors:** Clet Wandui Masiga

**Affiliations:** ^1^Tropical Institute of Development Innovations, Entebbe, Uganda

**Keywords:** biotechnology, biosafety, genetically modified, parliament, legislation, entrepreneur

## Abstract

The Cartagena Protocol on Biosafety to the Convention on Biological Diversity is an internationally binding instrument addressing issues of biosafety. Biosafety refers to the need to protect human health and the environment from the possible adverse effects of the products of modern biotechnology. Accordingly, all countries to the convention are required to put in place regulatory mechanisms to enhance the safety of biotechnology in the context of the Convention’s overall goal of reducing all potential threats to biological diversity, while taking into account the risks to human health. Therefore, each country party to the convention has its own procedures to enact laws to guide the safe use of biotechnology. In Uganda, the process involves the drafting of the bill by the first parliamentary counsel, approval by cabinet, first reading at the parliament, committal to the responsible parliamentary sessional committee, tabling of the bill for public hearing, consultations, and final approval. In Uganda, the Committee on Science and Technology is responsible for the Biosafety Bill. In March 2013, the Committee tabled the bill for public hearing and submissions from public institutions. There were comments supporting the passage of the Bill and comments in objection. The reasons for objection are mainly due to precaution, speculation, lack of knowledge about biotechnology and biosafety, and alleged influence from biosafety entrepreneurs. This article reviews these public views, revealing controversy and possible consensus to pass the bill.

## Introduction

The modification of plants and animals to obtain new ones with traits desired by farmers has been going on for millennia. It involved random recombination of DNA in offspring followed by selection for traits best suited for food, fiber, feed, and energy production (Barrows et al., 2014). This breeding was typically slow, sometimes limited by availability of desired traits in related species, and often took decades and frequently yielded crop varieties with unforeseen and undesirable properties. More recently, the process evolved to include the use of biotechnological techniques, particularly genetic engineering, to reduce uncertainty and breeding time and to transfer traits from more distantly related species (Andersson et al., 2014; James, 2014; Sharma et al., 2014). Biotechnology encompasses any technique that uses living organisms or substances from such organisms to make or modify a product, to improve plants or animals, or to develop micro-organisms for specific purposes (Masiga et al., 2014). Plants and animals bred through genetic engineering are normally referred to as genetically modified organisms (GMOs).

In some countries, the adoption of GMOs has occurred with little objection, whereas in others, there has been fierce controversy (Stone, 2010). The origin of this controversy seems to have been partly legitimized through international biosafety legal instruments, the Convention on Biological Diversity (CBD), and the Cartagena Protocol on Biosafety (CPB). The CPB required each signatory to put in place a national legal framework for safe development and commercialization of GMOs. As a consequence, most countries in Africa have either put in place, or are working to develop, legal frameworks for development and deployment of GMOs.

Each country party to the convention has its own procedures to enact laws. In Uganda, the process involves the drafting of the bill by the first parliamentary counsel, approval by cabinet, first reading at the parliament, committal to the responsible parliamentary sessional committee, tabling of the bill for public hearing, consultations, and final approval. The process for drafting the bill started in 1997 with the drafting of the National Biosafety Framework (NBF) to ensure safety in biotechnology. This was approved in March 2001 and in the same year the guidelines on biosafety in biotechnology were developed. This was followed with the establishment of the National Coordinating Committee (NCC) in 2002 to revise and publish biosafety guidelines, which was later followed by the drafting of the national policy on biotechnology and biosafety in 2003. The draft policy was later subjected to several consultations, reviews, and inputs from experts and in April 2008, it was approved by Cabinet. In the same year, the approved policy in turn led to the development of a draft Biosafety Bill. In March 2013, the bill was tabled by the parliamentary Committee on Science and Technology for public hearing and submissions from public institutions. The submissions were both for and against the bill.

This polarization of opinion indicated a need to better inform the public regarding the technology. This would help to clarify or amplify points of divergence (Juma, 2003). In 2013, to build capacity for Ugandans to support a bill, a world-renowned authority on the role of innovation in economic development Prof. Calestous Juma was invited to give a public lecture on the use of science and engineering for rapid economic transformation. In this article, I present views from the online submissions that lasted from 19th April 2013 to 8th October 2013 following the public lecture. The issues raised and their clarifications have parallels with those against and in favor of adoption of GMOs, except the facts on either side are often skewed to influence the debate in their own direction. Several of the “anti” positions are based on emotion, and may be countered by scientific literature or facts. The next section presents the main categories of arguments submitted.

## International Obligations

Those against the bill claimed that it does not conform to international obligations set out in the CPB (United Nations Environmental Programme (UNEP), 2000) and the CBD (United Nations Environmental Programme (UNEP), 1992). Those promoting it insist the bill has been prepared in response to the international obligations and has all relevant sections required by the protocol and the misinformation arises from the different interpretations of the clauses and deliberate twisting of facts. Clause 29 of the bill emphasizes safety in using biotechnology by providing for measures to be taken to minimize or avoid risk to human health and the environment arising from actual or potential contact with a GMO. Article 17 of the CPB requires Uganda to provide for emergency measures to deal with unintentional release of a GMO. Clause 30 gives effect to that obligation by requiring every application for research or general release to contain an emergency plan complete with safety measures to cater for circumstances where a GMO is released unintentionally to the environment. It is also important to note that the Protocol leaves significant flexibility for implementing instruments at the national level, as shown by many other countries (e.g., Kenya). Also, the scope of the Protocol deals with transboundary movement of living modified organisms (LMOs) only, whereas national laws have a more comprehensive scope. The argument whether a law does or does not follow the Protocol is always difficult to interpret.

## Scope of the Bill

Those against the bill claim that its scope is restrictive as it only applies to general releases of GMOs and does not mention the full range of activities involved. Those in favor insist that the bill is clear on these issues and is in line with article 4 of the Cartagena Protocol and therefore complies with the full range of activities, including research, contained use, confined field trials, import, export, and general release of a GMO (Government of Uganda, 2012).

## The Objective of the Bill

One of the objectives of the bill is to facilitate the safe development and application of biotechnology. Those against the bill have indicated that this objective is to facilitate and not regulate the introduction of GMOs in the country. If passed, it will create an enabling policy environment to promote GMOs in the country. Their intention is to restrict adoption of GMOs. Those for the bill indicate that the CPB recognizes “that biotechnology has great potential for human well-being if developed and used with adequate safety.” Their intension is to have a bill that facilitates the adoption of GMOs. As such the promoters of the bill argue that the objectives are in agreement with national agenda as well as meeting international obligation as envisaged in the CPB and should be done in a manner that does not disrupt trade (United Nations Environmental Programme (UNEP), 2000; Excellence Through Stewardship, 2015). There are many studies that currently favor the use of GMOs in increasing food, fuel, and fiber production (Wieczorek, 2003; Kwiecinski, 2009; Chipman, 2010; Jeanes, 2013; Kuntz et al., 2013; Nature, 2013).

## The Precautionary Principle

Those against the bill argue that the precautionary principle should prevail until sufficient evidence becomes available to prove that GMOs are safe. The promoters interpret the principle to mean that if you are not absolutely sure about the safety of a GMO, you can make a decision to use it. A review of the relevance of the precautionary principle in risk assessment confirms the divergent views about its application, which allows opposite conclusions depending on the context (Juma and Honca, 2002; Saner, 2002).

## Publication of the Application

The bill requires a notice in the prescribed form of the application for general release of the GMO to be published in the Gazette and the official website of the Competent Authority.

Opponents of the bill have indicated that after receipt of application, it should also be published in all local newspapers. Those promoting GMOs observe that the publication of the application follows other government procedures otherwise it will set a precedence that is against the constitution and other government laws.

## Expedited Review

The bill highlights circumstances when it is necessary for the Competent Authority to expedite the review of an application for research or general release of a GMO. Those against the bill argue that it provides for expedited review of an application where a competent authority of another country has previously approved the GMO in comparable ecosystems. They recommend that this provision should be stricken from the bill because there are no two ecosystems that are similar and that the risk of GMOs should be carried out on a case-by-case basis. Proponents argue that this provision is in line with the current harmonization of policies to facilitate regional trade.

## Liability and Redress

The bill provides for offenses and penalties to any person and corporate bodies. Those against the bill argue that it does not specifically address who will be responsible for the liability. They believe that the liability and redress system as provided for in the bill has been vaguely defined to give protection to the multinational corporations that will be promoting their technologies and ignore the rights of farmers. They also believe that the bill provides for a fault-based liability principle instead of a strict liability approach. Those in favor argued that liability and redress are well covered through other legal instruments. They also argue that strict liability is shallow, vague, envious, and a political argument and has been overtaken by time.

## Public Participation

Those against the bill argue that it does not provide an elaborate public participation mechanism. It only mandates that the competent authority promote awareness and does not specify the rights of the public to participate in the decision-making process. Those in favor of the bill have argued that scientific facts should not be subjected to a debate where one side must win and that, public participation is incorporated into the Biosafety Bill following international best practices.

## Labeling

Those against the bill argue that it does not have an explicit provision on labeling to allow for consumers to have a choice. Those in favor argue that the labeling is not necessary because it will either increase the cost of those farmers producing GMOs or traders of the GM foods. It will also be extremely difficult to enforce such a law as most agricultural trade in Uganda is informal and it is not easy to trace a product to one particular farmer.

## Patents and Rights

Those against the bill argue that GMOs will increase farmer costs because GM seeds are patented, which affects farmer practices to save, share, and multiply seed in interests of sustaining food systems. They believe that farmers’ right to save and replant saved seeds will be lost due to patented GMO seeds. Those in favor of GMOs argue that the costs associated with patents and rights would not affect costs to farmers who choose not to use the technology.

## Use of Publications and Pseudoscience

Both sides cited published literature that favored their view of the argument, and each side discounted the other’s choices of supporting documentation. For example, those against the bill have made good use of anti-GMO reports and publications to back their claims to resist GMOs. Those promoting GMOs believe that these negative publications are authored by biosafety entrepreneurs for business and career development.

Anti-GMO activists have relied on flawed publications to reject the bill. For example, they have used a publication reporting that rats fed on a lifelong diet of a common strain of genetically modified corn developed tumors and severe damage to their liver and kidneys (Séralini et al., 2012). A review of the publication indicated serious weakness of design, conduct, and analysis and was subsequently retracted (Séralini et al., 2014a,b). Another publication that is largely used to reject the bill is the one concluding that contrary to often-repeated claims that today’s genetically engineered (GE) crops reduce pesticide use, the spread of glyphosate-resistant weeds in herbicide-resistant weed management systems has brought about substantial increases in the number and volume of herbicides applied (Benbrook, 2012). Review of this paper showed that it was flawed being based on inaccurate claims, biased assumptions, and misleading use of official data (Brookes et al., 2012).

## Economic Argument

Rejection of the bill is also based on Schnurr (2013), who published that there is network of corporate actors, development agencies, policy officials, and research scientists that support the unquestioned dominance of GM in Uganda. GMO research and commercialization is driven by donors and has nothing to do with local demand (Schnurr and Gore, 2015). Those to the contrary argue that the technical and infrastructural capacity that has been built in Uganda is designed to enable Ugandans to develop and commercialize GMOs that are safe for humans, the environment, and biodiversity. They argue that it is false that farmers do not need GMOs and that these GM technologies are not demand driven. The approach used for GMO development and commercialization is not different from any other breeding technique in the country.

## Bribes by Multinationals

There is a perception that legislators, government civil servants, cabinet officials, scientists, academics, journalists, and any other person who see benefits in GMOs for mankind have been paid by Monsanto to market their products. Those promoting GMOs also argue that most of those against GMOs are receiving funding from the Environmental Grant Makers Association to block adoption of GMOs and are in it for business and interest of their funders.

## Capacity for GMO Development and Management

Anti-GMO groups believe that there is inadequate scientific knowledge within Africa. So, this particular scientific “adventure” is simply wrong. Therefore, Africa is not ready at all for these GMOs, given African infrastructure, technology, literacy levels, capacity in terms of risk assessment, environmental protection, etc. Those supporting the bill argued that Uganda has built significant human and infrastructural capacity to handle GMOs. There are more than 140 Ugandan scientists working in agricultural biotechnology and more than 15 Ugandan institutions conducting biotechnology research, including public and privately owned businesses (International Food Policy Research Institute (IFPRI), 2013).

## Unexpected Resistance to Herbicides and Emergence of Superweeds

Those against the bill argue that GMOs have led to unexpected resistance to herbicides and emergence of superweeds plus the huge expenses associated with managing the superweeds as a result of herbicide tolerant technology. Promoters, on the other hand, argue that this is a natural trend and not specific to GMOs. There are four historical and biological examples that were used to illustrate this point, which include the story of industrial melanism in England involving the peppered moth (Kettlewell, 1955), resistance to antibiotics, fungicides/insecticides, and heavy metal resistance in plants.

## Conspiracy Theories

Those against the bill believe that GMO promoters are under a conspiracy to shorten the life span of Africans. So unlike in Africa, where GMOs will be consumed directly, GM corn produced in the US and other countries is converted into high-fructose corn syrup or used in other industrial processes that break down GE crop components into ethanol/biodiesel and vegetable oils or fed to livestock. GMO proponents argue that there are a number of credible studies done on the safety of GMOs that have indicated that there is no significant difference between the safety of GMOs and non-GMOs.

## Market for Farm Produce

Some GMO opponents are worried that Uganda may lose market access for their farm produce if the country adopts GMOs. They believe that foreign markets that are uninterested in the GMOs will not buy farm produce from countries growing GMOs, hence resulting in a significant loss for the farmers. To the contrary, those in favor of GMOs believe that Europe, which has been the main block against GMOs, imports a lot of food from countries that grow GMOs.

## Main Agricultural Constraint is Not Production

Those against the bill believe that the problem facing farming in Uganda is not production but other constraints, such as post-harvest handling, processing, and distribution. The proponents believe that GMOs are used to target specific traits in response to specific challenges, particularly those that have not been possible to address using conventional means.

## Conclusion and Way Forward

There have been, and will continue to be, public debate about GMOs. But considering the submissions from the two sides of the arguments, the bill is clear on its objectives and it conforms to the requirements of the international obligations, as the crux of the argument for going forward. The Bill as it is neither promotes nor prohibits the technology, but it ensures that it will be used only when regulators determine it is safe. Both sides of the argument should be able to support that. A law that addresses all concerns of the people is very unlikely. And as such the most feasible option is to take it as it is and then revise it as the country uses it. In its current state, the bill appears to allow the adoption of GMOs while ensuring that they are safe to humans, biodiversity, and the environment.

## Conflict of Interest Statement

The author declares that the research was conducted in the absence of any commercial or financial relationships that could be construed as a potential conflict of interest.
